# Developing English language learners’ oral production with a digital game-based mobile application

**DOI:** 10.1371/journal.pone.0232671

**Published:** 2021-01-05

**Authors:** Zehua Wang, Feifei Han

**Affiliations:** 1 School of Foreign Languages, Shaanxi Xueqian Normal University, Xi'an, Shaanxi, China; 2 Office of Pro-Vice-Chancellor (Arts, Education and Law), Griffith University, Brisbane, QLD, Australia; 3 Griffith Institute for Educational Research, Griffith University, Brisbane, QLD, Australia; Fondazione Ugo Bordoni, ITALY

## Abstract

This study examined the effect of using a digital game-based language learning mobile application “Liulishuo” (speaking English fluently) to develop complexity, accuracy, and fluency of English monologic oral production among 30 English language learners in China. Monologic oral production was measured using the same narrative picture description task in pre- and post-tests. The learners followed the “Imitation of English Monologues” game 30 minutes each time, twice a week, for 20 weeks. The oral production was measured using six indices: the mean words per T-unit and lexical density (i.e., complexity), the mean repairs and errors per 100 words (i.e., accuracy), speech rate and the mean length of pauses (i.e., fluency). The paired sample *t*-tests showed that the participants produced more complex monologic speech, had significantly fewer errors, and increased speech rate, but the mean repairs and mean length of pauses remained unchanged. The unchanged repairs and pauses could be possibly due to the non-proceduralized linguistic knowledge in oral production, which may require a more extended period of treatment. Our study showed positive effects of using a digital game-based language learning mobile application on the improvement of complexity, accuracy, and fluency of English language learners in China’ monologic oral production with varying effects.

## 1. Introduction

Technology has undoubtedly opened a new era of human being’s experience in every domain, including students’ learning experience in higher education. With the assistance of technology-enhanced learning, students have more freedom than before to make decisions as to when, where, and how to learn. This study reports the use of a digital game-based mobile application in learning English as a foreign language classroom to develop English language learners’ oral production in one academic semester. The study measures the changes in oral production in terms of complexity, accuracy, and fluency between the pre-test, which was held at the beginning of the semester, and the post-test, which was held at the end of the semester after 20-week using of the mobile application.

### 1.1 Digital game-based learning

In recent decades, digital games have been increasingly integrated into technology as an instructional tool in teaching and learning, known as digital game-based learning. [1] defines the game as: “a system in which players engage in an artificial conflict, defined by rules, that results in a quantifiable outcome” (p. 80). Digital-game based learning can make learning more interactive, authentic, exciting, stimulating, and well-structured than traditional ways of learning [2–5].

Digital-game based learning has been applied in different fields, such as in education, defense force, marketing, governmental organizations, and health and wellbeing sectors [6]. The games focusing on learning new knowledge tend to be adopted in science, technology, engineering, and mathematics education, and language learning [7]; whereas the games targeting skill development are predominantly implemented in military and corporate contexts [8].

The accumulated evidence has revealed the benefits of adopting digital-game based learning in various settings, such as in air force and navy technical training [9, 10] and science, technology, engineering, and mathematics education [11–14]. Compared with the amount of research on digital-game based learning in science, technology, engineering, and mathematics education, research on digital-game based learning in language education and learning is comparatively less [15, 16]. The following literature discusses the use of digital games to learn a foreign language (known as digital game-based language learning).

### 1.2 Digital game-based language learning

An increasing number of educators have recognized how digital games can be pedagogically exploited to facilitate language acquisition in recent years [17]. Digital game-based language learning is “the implementation of computer [games] that include an identifiable teaching presence especially for improving some aspect of language proficiency” [18]. With a belief that digital game-based language learning can enhance learners’ intrinsic motivation and enjoyment in language learning, more and more teachers have employed various digital games in foreign language classrooms [19].

In a special issue on digital game-based language learning research, [15] identified five major research themes, namely (1) theory development theme, which integrates concepts of digital-game based learning and foreign language acquisition theories; (2) design theme, which evaluates technological aspects of one or a digital games in language learning and teaching; (3) pedagogical theme, which is concerned with teachers’ self-reflection of the effectiveness of using certain digital games in language teaching; (4) experimental studies, which compare the effect of digital game-based language learning intervention with a control group; and (5) non-experimental empirical studies, such as case studies, which report the effects of using digital game-based language learning to develop one or more skills in foreign language learning. Our research fits into the last theme because the primary purpose of our study was to use digital game-based language learning as an aid to develop learners’ oral production. Studies on digital game-based language learning have particularly targeted on the development of vocabulary [20] than the other aspects of language acquisition. In particular, there is a dearth of studies that examine using digital games to develop foreign language oral production, which is the focus of our study. In our study, the digital games we used are mobile-based because mobile applications have gained increasing popularity in recent years.

### 1.3 Measurement of foreign language production

In the literature of foreign language learning, it is widely acknowledged that foreign language production is multidimensional in nature, hence, it is difficult to evaluate it using a single measurement [21–23]. Two main frameworks have been proposed and testified in an attempt to represent foreign language production. One is the Common European Framework of Reference for Languages, which was constructed and developed by [24]. The Common European Framework of Reference for Languages uses communicative competence construct to represent foreign language production along a continuum in a number of categories and sub-categories. The framework states in a detailed and qualitative manner what constitutes different levels of competence in terms of what a learner “can do” in foreign language reading, writing, listening, and speaking.

Another framework is well acknowledged as complexity, accuracy, and fluency triad. Departing from a componential perspective, the complexity, accuracy, and fluency triad measures foreign language production using multiple indicators for each of the three aspects [21, 25, 26]. One of the major advantages of using the complexity, accuracy, and fluency triad is advantageous over the Common European Framework of Reference for Languages to benchmark and assess foreign language production as it employs multiple indicators underlying each aspect [27]. In most cases, these indicators are quantifiable measures, which make it easier to compare production between learners and/or to trace the development of production over time. Hence, the complexity, accuracy, and fluency triad is widely adopted in longitudinal studies to describe and assess foreign language learners’ written and oral production [21, 25, 28, 29]. As our study examined the development of learners’ oral production in pre- and post-test, using the complexity, accuracy, and fluency triad is more appropriate.

The indicators in the complexity, accuracy, and fluency triad are quantifiable measures, which make it easier to compare production between learners and/or to trace the development of production over time.

The first component in the complexity, accuracy, and fluency triad is complexity, which represents the breadth and depth of language production [30]. Complexity is comprised of different language features at different levels, ranging from the sub-lexical level, such as phonological and morphological complexity, to word-level, such as lexical complexity, and the above word-level, such as syntactic complexity [31]. While the sub-lexical complexity is often regarded as trivial, thus does not attract much research; the lexical and syntactic complexity are frequently used in measuring foreign language production [32, 33]. Therefore, we used lexical and syntactic complexity as indicators of complexity. For lexical complexity, we used lexical density; and for syntactic complexity, we used the average number of words per T-unit (see coding for details).

The second component in the complexity, accuracy, and fluency triad is accuracy, which describes the level of conformity of language production to certain norms [23]. This dimension reflects the level of target-like use of the language [34]. There are two ways to represent the accuracy, one examines whether a learner supplies a linguistic form in obligatory contexts, which is suitable for measuring accuracy of a specific linguistic form. Focusing on general accuracy, the other means examines the numbers of errors in a wide range of linguistic features [34]. As our study does not focus on one particular linguistic form, we employed the general accuracy. We used the average number of repairs and errors per 100 words to represent the accuracy dimension (see coding for details).

The third dimension in the complexity, accuracy, and fluency triad is fluency, which reflects the extent of the automaticity of language production [35]. Fluency can be measured by two different approaches: one is related to the length of production and the other concerns with the time of production. While the length approach is often favored in measuring written production, the time is preferred in assessing oral production [36, 37]. Therefore, we adopted time-based measurements, which included speech and pause rate (see coding for details).

### 1.4 The present study

The present study aimed to examine the effect of using one of the most popular digital game-based language learning mobile applications–“Liulishuo”, which is specifically designed for English language learners in China, on the development of English monologic oral production measured using the complexity, accuracy, and fluency triad in a pre- and post-test after 20-weeks’ use of the application.

The present study addressed the following three research questions:

1. To what extent did the complexity of English monologic oral production differ between pre- and post-test?
2. To what extent did the accuracy of English monologic oral production differ between pre- and post-test?
3. To what extent did the fluency of English monologic oral production differ between pre- and post-test?

## 2. Materials and methods

### 2.1 Participants

The participants were 30 second-year undergraduate students enrolled in a four-year Bachelor’s degree in English Education in Early Childhood at a national university in China. All the participants were females as most of the early childhood educators in China are females. The participants’ ages were between 19 and 21 with a *Mean* (*M*) of 20. All the students started to learn English from grade three in primary school. At the time of the study, they had learned English for approximately 10 years.

### 2.2 Research design

The study adopted a longitudinal within-subject design. The English oral product of the same participant was traced for one semester and measured twice in a pre-test at the beginning of an academic semester and in a post-test at the end of the academic semester.

### 2.3 Materials

#### 2.3.1 Monologic narrative picture description task

We used a monologic narrative picture description task to elicit the participants’ oral production for two reasons. First, monologic tasks are free of influence by international factors as those in the dialogical tasks, which are heavily impacted by the performance of interlocutors and the listening proficiency of learners [38]. Therefore, compared with dialogical tasks, monologic tasks are more representative of students’ oral proficiency. Second, we purposefully selected the narrative genre because the participants had an intensive practice of narratives in English oral production as indicated by their English teacher. Moreover, narratives require less topical knowledge compared with expositions [39]. We used the same picture description task to measure the pre- and post- oral production because this reduced the variations of task difficulty to the minimal level, which was able to reflect the development of learners’ monologic oral production. Due to the long period between the pre- and post-tests (20 weeks), the influence of task repetition would be negligible.

The task required the participants to describe a series of six pictures in English by linking them in a story. The first picture presents a scene that a little girl, her parents, and their dog are going to a farm. The second picture depicts that while the little girl and the dog are playing together at the bank of a river near the farm, her parents are busy doing farming work. The third picture describes an accident that the little girl falls into the river. In the fourth picture, the dog is barking loudly on the bank of the river and the parents are looking at the dog. In the subsequent picture, the dog is in the river and is trying hard to push the girl to the bank. In the last picture, the girl is safe and the couple is so grateful to the dog for saving the girl.

We piloted the picture description task with five students with a similar background and English proficiency to ensure that there would be no difficulty in understanding the pictures and describing them in English. The feedback from these students suggested that the task was appropriate for our participants.

#### 2.3.2 Digital game-based language learning mobile application

We used “Liulishuo–A Speaking Tutor, Grading Your Spoken English” mobile application as an aid to develop the learners’ monologic oral production. “Liulishuo” which means “speaking English fluently”, is developed by Shanghai Liulishuo Information Technology Limited. It has 720 million users (as shown by 2018 statistics), and ranked as one of the most popular mobile applications in practicing spoken English among English language learners in China. The participants were required to follow “Imitation of English Monologues” game 30 minutes twice a week to practice monologic oral English.

The application uses the largest spoken corpora of English speech of English language learners in China created and maintained by the company. The application utilizes a cutting-edge speech recognition system to recognize English speech produced by English language learners in China. It allows users to practice spoken English via mobile microphones and can record learners’ speech, which is then analyzed using sophisticated algorithms integrated with the application to compute a score for learners’ oral production as immediate feedback. The application assesses the oral production using seven major indicators: (1) pronunciation, (2) stress, (3) intonation, (4) grammatical accuracy, (5) appropriateness of pragmatics, (6) coherence, and (7) fluency. The application can be installed into both IOS and Android systems.

The application has a number of unique features. First, it can stimulate learners’ interests and passion to practice spoken English through game-based elements, such as rewarding users with gold coins and stars, ranking users, simulating competitions among game players, and allowing users to break through into different stages like those in other digital games. Second, the topics in the practice units are diverse so that they can satisfy users from different age groups, educational and occupational backgrounds, and with different interests and hobbies. Third, the automated scoring system and the individualized dashboard sustain learners’ motivation and help them set achievable goals in different stages of learning.

### 2.4 Data collection procedure

Before the data collection, an ethical application was submitted to the ethics committee of the School of Foreign Languages, Shaanxi Xueqian Normal University. The committee evaluated the study and deemed that the research would be part of classroom teaching. Hence, a written consent procedure was not required. However, all participants orally agreed to take part in the research voluntarily. “All participants orally agreed to take part in the research voluntarily” was documented by the ethics committee and the ethics committee approved the study.

The study adopted a longitudinal within-subject design, which involved a pre-test and post-test. At the beginning of the semester, the researchers explained to the students the purposes of the study and invited them to voluntarily participate in the study. The pre-test (1^st^ week of the semester) conducted individually measured the participants’ initial level of monologic oral production. After the pre-test, each student was instructed individually on how to install the mobile application and on what she needed to do for the practice in 20 weeks. During the 20 weeks, they were required to record their practice on their mobiles and the recordings were regularly checked by one of the researchers to make sure that they followed the requirements. After 20-week, they narrated the same pictures in the post-test. Both pre- and post-tests were conducted in a quiet office, which ensured the quality of audio-recordings of the students’ oral production.

### 2.5 Coding and data analysis

The audio-recordings were transcribed. The transcripts and the audio-files were coded in terms of complexity, accuracy, and fluency. As mentioned, we examined syntactic and lexical complexity. We used the mean words per T-unit for syntactic complexity rather than using other more complex measures, such as the number of independent, coordinate, or subordinate clauses, which are predominantly used for assessing written scripts [40], [22]. We calculated the mean words per T-unit using Sentence Extractor (http://www.lextutor.ca/tools/ex_sent/). A T-unit is an independent clause and any dependent (subordinate) clauses or non-clausal structures that are attached to or embedded within it [41]. More mean words per T-unit indicates higher syntactic complexity. To examine lexical complexity, we used lexical density, which is defined as the ratio of the number of lexical words to total words of learners’ production [42]. The higher value of lexical density suggests a higher level of lexical complexity of the speech.

To examine accuracy, we used mean repairs per 100 words and mean errors per 100 words. The mean repairs per 100 words were calculated by dividing the total number of repairs by the total number of words in the oral production and then multiplied by 100. According to [43], there are five types of repair, including reformulation, replacement, repetition, false start, and hesitation. Reformulation is defined as repeating a phrase or a clause by modifying syntactic features of the phrase or the clause (e.g., The girl **see**…The girl **saw** a ball). Replacement refers to substituting a phrase or a clause with another phrase or a clause (e.g., The girl is **following**…**chasing** the dog). Repetition is restating the same phrase or clause without any modification (e.g., The girl…**The girl** is angry). False start means completely giving up a phrase or a clause at the beginning of a sentence and then restarting using another phrase or a clause (e.g., **The girl is**…**Her parents are** waving to her). Hesitation is referred to as the repetition of a phoneme or a syllable within a word (e.g., The dog **ba…** barked to her parents).

The mean errors per 100 words were calculated by dividing the total number of errors by the total number of words in the speech and multiplied by 100. We included both syntactic errors (e.g., Her parents is (are) working on the farm) and lexical errors (e.g., The girl is catching (picking) the flower). The fewer the mean repairs and mean errors per 100 words suggest the more accurate the oral production is.

To examine fluency, we coded the speech rate and the mean length of pauses under the assistance of the software Cool Edit Professional 2.0 using the audio-files. The speech rate was expressed in terms of the number of words per minute and was calculated by the total number of words divided by the speech length (in minutes). A higher value of the speech rate represents more fluent speech. The mean length of pauses was expressed in seconds and was calculated by averaging the length of all the pauses in a speech. The longer mean length of pauses means less fluent speech. In our study, a pause was identified as a break of one second or longer either within a sentence or between sentences. The length of one second was considered appropriate by taking into consideration the participants’ English oral proficiency. We entered the coded data into IBM SPSS 22 and conducted paired sample *t*-tests to examine if there were differences between pre- and post-test.

## 3. Results

Table 1 presents descriptive statistics and the results of paired sample *t*-tests. The paired sample *t*-tests showed that in terms of the complexity measure, the **mean** words were significantly more in the post-test (*M* = 15.56, *SD* = 4.12) than in the pre-test (*M* = 11.87, *SD* = 2.94), *t* (1, 29) = -4.86, *p* < .01. This suggests that in the same amount of time, the learners produced more English words in a clause after 20 weeks’ using the mobile application. Likewise, we also observed that the lexical density in the post-test (*M* = 0.50, *SD* = 0.07) was significantly higher than that in the pre-test (*M* = 0.46, *SD* = 0.06), *t* (1, 29) = -2.24, *p* < .05.

**Table 1:** Descriptive statistics and the results of paired sample *t*-tests.

triad	indices	pre-test		post-test		*t*	*p*
		*M*	*SD*	*M*	*SD*		
complexity	mean words per T-unit	11.87	2.94	15.56	4.12	-4.86	.00
	lexical density	0.46	0.06	0.50	0.07	-2.24	.03
accuracy	repairs per 100 words	6.16	3.77	5.51	2.33	1.00	.33
	errors per 100 words	8.23	2.74	5.98	2.73	4.36	.00
fluency	speech rate (per minute)	45.99	14.37	60.84	15.40	-5.11	.00
	mean length of pauses (second)	3.73	2.09	3.09	2.14	1.21	.24

With regard to the two indices of accuracy, we found that while the number of repairs per 100 words remained unchanged between the pre- (*M* = 6.16, *SD* = 3.77) and post- oral production (*M* = 5.51, *SD* = 2.33), *t* (1, 29) = 1.00, *p* = .33; the participants had significantly fewer errors, hence were more accurate, in the post-production (*M* = 5.98, *SD* = 2.73) than in the pre-production (*M* = 8.23, *SD* = 2.74), *t* (1, 29) = 4.36, *p* < .01.

For fluency, we observed that while students’ speech rate improved significantly between the pre- (*M* = 45.99, *SD* = 14.37) and post-tests (*M* = 60.84, *SD* = 15.40), *t* (1, 29) = -5.11, *p* < .01; the mean length of pauses did not change significantly between the pre- (*M* = 3.73, *SD* = 2.09) and post-tests (*M* = 3.09, *SD* = 2.14), *t* (1, 29) = 1.21, *p* = .24.

## 4. Discussion

The study examined the effect of one of the most popular digital game-based language learning mobile applications “Liulishuo” in China on Chinese university English language learners’ monologic oral production measured by the complexity, accuracy, and fluency triad between pre- and post-tests in 20 weeks. For complexity, we found that after 20-week practice using the digital game-based language learning mobile application, the spoken speech of describing a story in English generated by our learners not only had more words in a clause but also had more content words than what they could do at the beginning of the semester. Our results corroborated previous research, which also demonstrated the positive effect of using mobile devices in language learning, albeit in other skills, such as writing and listening skills [44, 45]. Speaking is a complex skill whose development not only requires ample input and but also requires repeated output [46], digital game-based mobile application can provide students with both visual and audio input via game elements and flexible delivery, which are likely to sustain learners’ efforts.

In terms of accuracy, we found that the participants had significantly reduced error rates in terms of grammar. We found that the participants were more accurate in terms of subject-verb agreement, used tenses more consistently, used correct sentence structures and propositions following verbs. They were also able to be more accurate in terms of using correct lexical items to convey the meaning. However, the participants did not reduce their repair rate in the monologic speech, which could be possibly due to that the participants had not yet proceduralized the declarative form of grammatical and lexical knowledge. Hence, in concurrent production tasks, such as oral production, which requires relevant knowledge, being grammar and/or vocabulary to be retrieved rapidly online and held in the working memory [47]. We speculated that our participants might be still slow at retrieving relevant linguistic sources from their long-term memory. Hence, they might have made mistakes in the initial attempt to form a sentence in speech using linguistic knowledge and repaired these mistakes by themselves subsequently. This was also related to the context of English learning of our participants because they mainly learned English in formal classroom settings through direct and explicit instruction. Such a learning context seemed to suggest that it takes longer for the learned declarative linguistic knowledge to be proceduralized and for the repair rates to be lowered. One possible way to testify this speculation is to extend the treatment period (e.g., one year) or to intensify the treatment (e.g., using the mobile application five times per week).

With regard to fluency, we found that only speech rate was improved significantly between the pre-test and post-test, and this indicated that our learners were able to narrate the same story in English faster after 20 weeks using the “Liulishuo” mobile application. However, the average length of pauses remained unchanged. We speculated that such non-change could indicate that our participants still used much cognitive effort in English oral production. Hence the time required for them to convert concepts into English linguistic codes did not change much. Another possible interpretation could be the translation strategies used by our participants in English oral production from casual interviews with them. Due to a lack of practice in spoken English, the participants tended to form sentences in Chinese first and then translated into English, which were likely to result in long pauses.

In general, our study showed a positive effect of using “Liulishuo”–a digital game-based language learning mobile application on the development of Chinese English language learners’ English monologic oral production on complexity, accuracy, and fluency aspects, albeit the degree of the development of the three aspects varied. As Chinese students learn English mostly in classroom setting, which does not offer sufficient input and output, apart from practice they do as requirements in English classes, they also need to be supplied with other opportunities. In this regard, digital game-based mobile application for speaking enable students to practice spoken English in their free time and their preferred locations. Hence, it can be employed as an effective educational aid and a supplementary source to extend students’ formal learning experience to informal settings through diverse leaning activities [48–51]. The overall findings of this study corroborate the results of previous research, which also demonstrated that language learners produced more meaningful sentences and their speech was more accurate and fluent after a sustained period of using digital game-based mobile learning tools (e.g., [44, 45, 48]). Our study suggests a potential for such mobile applications or similar kinds to be integrated into spoken English instruction.

## 5. Conclusions

Our study made a preliminary attempt to investigate the effect of using a popular mobile application in foreign language monologic oral production development. The study is small scale and suffers from a number of limitations, which we will address in our follow-up studies. First and foremost, our study was not a true experimental study because our aim was to improve students’ oral production in English. To extend our study, we plan to compare the development of oral production of three groups of students using an experimental design. One group of students will receive the digital game-based language learning treatment using the mobile application, another group will receive the traditional ways of developing spoken English, such as oral presentation skills training [52], and the third group of students will serve as a control group.

Secondly, in the follow-up studies, we plan to include more indicators for each of the complexity, accuracy, and fluency aspects. The inclusion of more indicators will allow us to discover fine-grained development, which may not be reflected in the indicators covered in the present study. For instance, to measure lexical complexity, we will use lexical diversity and lexical sophistication. The former can be calculated by dividing the number of word types by the total number of words, and the latter can be obtained by dividing the total number of sophisticated words per total number of lexical words [53, 54].

Thirdly, our study investigated solely the effect of the digital game-based language learning mobile application on the learning outcomes, without considering if the application has some effects in the learning process, such as motivation, attitudes, and enjoyment in the learning [55]. Based on the present study, we will also compare students’ motivation and enjoyment in developing English oral production between using the digital game-based language learning mobile application and using oral presentation training methods.

## Supporting information

S1 Data (ZIP)
